# Dual roles of suberin deposition at the endodermal Casparian strip in manganese uptake of rice

**DOI:** 10.1093/jxb/eraf302

**Published:** 2025-07-07

**Authors:** Toshiki Fujii, Naoki Yamaji, Jian Feng Ma

**Affiliations:** Institute of Plant Science and Resources, Okayama University, Chuo 2-20-1, Kurashiki 710-0046, Japan; Institute of Plant Science and Resources, Okayama University, Chuo 2-20-1, Kurashiki 710-0046, Japan; Institute of Plant Science and Resources, Okayama University, Chuo 2-20-1, Kurashiki 710-0046, Japan; University of Porto, Portugal

**Keywords:** Casparian strip, endodermis, manganese transporter, rice, root, suberin deposition

## Abstract

Rice roots are characterized by having two Casparian strips (CSs) at the exodermis and endodermis, where transporters for mineral nutrients are expressed. However, the exact role of the CS in expression of the transporters and subsequent nutrient uptake is poorly understood. Here, we first investigated the role of the CS in manganese (Mn) uptake by using a rice mutant (*oscasp1*) defective in formation of the endodermal CS. Knockout of *OsCASP1* resulted in decreased Mn uptake under limited Mn conditions, but increased Mn uptake at high Mn concentration. Immunostaining revealed that knockout of *OsCASP1* did not affect the cell specificity of localization of two transporters (OsNramp5 and OsMTP9) required for Mn uptake, but decreased the protein abundance of these transporters at the endodermis regardless of Mn concentrations tested. Furthermore, we found that overaccumulation of suberin at the endodermis of the mutants suppressed the expression of two transporters; the expression of the two transporters was only observed in the endodermal cells without suberin deposition, but not in the cells with suberin deposition. Taken together, our results indicate that there are two roles for the CS in Mn uptake; maintaining normal expression of the transporters at limited Mn concentration and preventing Mn diffusion to the stele at high Mn concentration.

## Teaser

The rice endodermal Casparian strip maintains normal expression of the transporters for Mn uptake at limited Mn concentration and prevents Mn diffusion to the stele at high Mn concentration.

## Introduction

To prevent uncontrolled diffusion of mineral elements including essential and toxic elements and microorganisms into the root stele, vascular plants have developed a barrier in the roots—the Casparian strip (CS) (Robbins *et al.*, 2014). Some plants such as Arabidopsis have one CS at the endodermal cells, while other plants such as rice have two CSs, one each at the exodermal and endodermal cells in the roots (Enstone *et al*., 2002). The exodermis and endodermis share similar structures, but they are not completely identical; exodermis differentiation occurs farther from the meristem and is often influenced by physiological conditions. In addition, CSs are broader in the exodermis and formed when suberin lamellae are also deposited (Geldner, 2013). Although the feature of the CS at the exodermal cells is still poorly understood, the CS at endodermal cells is a ring-like lignin structure deposited between endodermal cells to seal the apoplastic space, thereby creating a diffusion barrier (Naseer *et al.*, 2012; Geldner, 2013). The CS is also thought to play an important role in blocking leakage of mineral nutrients from the roots back to the soil (Shukla *et al.*, 2021). This role is especially important for rice because rice roots have a developed aerenchyma between the exodermis and endodermis (Enstone *et al.*, 2002). During the last decades, a number of genes involved in CS formation have been identified. In Arabidopsis, Casparian strip domain proteins (AtCASPs) were found to be involved in assembling lignin-polymerizing proteins including enhanced suberin 1 (ESB1), dirigent proteins (DPs), respiratory burst oxidase homolog F (RBOHF), and peroxidase 64 (PER64) (Roppolo *et al.*, 2011; Hosmani *et al.*, 2013; Lee *et al.*, 2013). A transcription factor, AtMYB36, was found to regulate ESB1, CASPs, and PER64 (Kamiya *et al.*, 2015; Liberman *et al.*, 2015), while CS integrity is controlled by the CS integrity 1/2 (CIF1/2)–Schengen 3 (SGN3)–SGN1 signal pathway (Doblas *et al.*, 2017; Nakayama *et al.*, 2017). In rice, a similar mechanism for CS formation was also reported (Wang *et al.*, 2019, 2022; Zhang *et al.*, 2023), although some difference between Arabidopsis and rice in the CIF1/2–SGN3 pathway was found (Zhang *et al.*, 2023). Recently, a new family of proteins, GAPLESS, was found to be required for tethering of CS membrane domains in rice (Song *et al.*, 2023).

On the other hand, the role of the endodermal CS in the uptake of mineral elements has been studied in several mutants of Arabidopsis and rice that are defective in the formation of the CS. However, the results differ with plant species, mutants, elements, and experimental conditions. For example, knockout of *AtCASP1* and *AtCASP3* in Arabidopsis decreased Ca accumulation in the shoots (Hosmani *et al.*, 2013), whereas knockout of *OsCASP1* in rice resulted in increased Ca accumulation in the shoots (Wang *et al.*, 2019). The *atmyb36* mutant accumulated more Mg and Zn in the shoots, but less Ca, Mn, Fe, and B compared with its wild type (WT) (Kamiya *et al.*, 2015). By contrast, a triple mutant of *OsMYB36a*, *b*, and *c* showed higher Ca levels and lower Mn, Fe, Zn, Cu, and Cd levels in shoots (Wang *et al.*, 2022). Even for the same mutants of *atsgn3* and *atsgn4*, more Zn and less K were found in the mutants compared with the WT in one experiment, but not in another experiment (Muro *et al.*, 2023). These differences in ionomic profiles between different plant species and mutants have been attributed to the root structure, uptake site, early suberization, etc. (Wang *et al.*, 2019 , 2022). However, the exact mechanisms underlying CS defect-induced ionome changes are poorly understood.

In the present study, firstly, we investigated the role of the endodermal CS in Mn uptake under different Mn concentrations using rice *oscasp1* mutants.

*OsCASP1* is required for CS formation at the endodermis of rice roots, but it does not affect the CS formation at the exodermis (Wang *et al.*, 2019). Knockout of *OsCASP1* resulted in decreased Mn accumulation under normal growth conditions (0.5 µM Mn) (Wang *et al.*, 2019), but the role of the CS under different Mn conditions remains unknown. We found that the CS plays different roles in Mn accumulation at Mn-limited concentrations and Mn-excess concentrations. Secondly, we examined the effect of a CS defect on the expression of transporters involved in Mn uptake. Mn uptake in rice is mediated by two transporters—OsNramp5 and OsMTP9—which are polarly localized at the exodermis and endodermis (Sasaki *et al.*, 2012; Ueno *et al.*, 2015; Shao *et al.*, 2017). We revealed that overdeposition of suberin in the endodermis of the *OsCASP1* mutant suppresses the expression of these transporters for Mn uptake.

## Materials and methods

### Plant materials and growth conditions

WT rice (*Oryza sativa* cv. Nipponbare) and two independent knockout lines of the *OsCASP1* gene reported before (Wang *et al*., 2019) were used in this study. All the seeds were soaked in deionized water for 2 d in the dark at 30 °C and then placed on a net floating on a solution containing 0.5 mM CaCl_2_. After growth for 2 d at 30 °C, seedlings were transferred to a 1.2 liter plastic pot containing half-strength Kimura B solution (pH 5.6) as described previously by Yamaji and Ma (2007) for various experiments. The nutrient solution was changed every 2 d. The plants were grown in a greenhouse under natural light conditions at 25–30 °C. At least three biological replicates were made for each experiment.

### Growth at different Mn concentrations

To investigate the effect of the endodermal CS on Mn uptake, two *OsCASP1* knockout mutants (*oscasp1-1* and *oscasp1-2*) and the WT (15 d old) were grown in half-strength Kimura B solution with different Mn concentrations (0.05, 0.5, 5, 50, and 500 µM). After 16 d, the SPAD values of the fully expanded leaves (leaf 5) were measured with a chlorophyll meter (SPAD-502 Plus; Konica Minolta, Japan). Before harvest, the plants were exposed to a solution containing 1 µM Sr, Rb, Ge, and Cd for 1 d. The roots were washed with 5 mM cold CaCl_2_ solution three times before harvesting, and the roots and shoots were separated using a razor. The root and shoot samples were subjected to Mn determination by inductively coupled plasma-MS (ICP-MS) as described below.

### Collection of xylem sap

Seedlings (40 d old) of both WT and two *oscasp1* mutants were exposed to a half-strength Kimura B solution containing different Mn concentrations (0.5, 50, and 500 µM). After 6 h, the shoots (2 cm above the roots) were excised with a razor, and then the xylem sap was collected with a micropipette for 30 min after decapitation of the shoot. The Mn concentration of the xylem sap was determined as described below after being diluted with 5% HNO_3_.

### Determination of mineral element concentration and root uptake

Plant samples harvested were dried at 70 °C for at least 2 d, and then digested by 61% HNO_3_ (w/v) as described previously (Huang *et al.*, 2020). The concentration of mineral elements in the digestion solution and xylem sap was determined with an ICP-mass spectrometer (7700X; Agilent Technologies, USA). Root uptake was calculated as follows: (shoot Mn content+root Mn content)/root DW.

### RNA extraction and gene expression analysis

To compare the expression of *OsNRAMP5* and *OsMTP9* in the roots between the WT and *oscasp1* mutants, RNA was extracted from seedlings (14 d old) grown in half-strength Kimura B solution. Total RNA was extracted using an RNeasy Plant Mini Kit (Qiagen, USA), followed by converting it to cDNA using the ReverTra Ace RT Master Mix with gDNA remover (TOYOBO; Japan) according to the protocol of the manufacturer. The expression of *OsNRAMP5* and *OsMTP9* was determined by quantitative real-time PCR (RT-PCR) using a Thunderbird SYBR qPCR mix (Toyobo) on a CFX96 PCR detection system (Bio-Rad, USA). The primers used were the same as reported previously (Sasaki *et al.*, 2012 ; Ueno *et al.*, 2015). *Histone H3* was used as an internal control. The relative gene expression was normalized by the ΔΔCt method using the CFX Manager software (Bio-Rad, USA).

### Immunostaining and quantitative analysis of protein level

Immunostaining was performed to observe the localization and protein levels of OsNramp5 and OsMTP9 in roots of the *oscasp1* mutants and the WT. Both the *oscasp1* mutants and the WT (6 d old) were exposed to a 0.5 mM CaCl_2_ solution containing different Mn concentrations (0.5, 50, and 500 µM). After 6 h, the seminal roots were sampled for immunostaining with OsNRAMP5 and OsMTP9 antibodies used previously as primary antibodies (Sasaki *et al.*, 2012; Ueno *et al.*, 2015), according to Yamaji and Ma (2007). Fluorescence of the secondary antibody (Alexa Fluor 555 goat anti-rabbit IgG; Invitrogen, USA) was observed using 561–580 nm for OsNramp5 and 561–575 nm for OsMTP9 with a confocal laser scanning microscope TCS SP8x (Leica Microsystems). The images used for quantification as described below were taken under the same conditions.

The signal intensity of the secondary antibody at the exodermis and endodermis was quantified using the draw polyline quantification tool of LAS X 3D software (Leica Microsystems) (Konishi *et al.*, 2023; Huang *et al.*, 2024). Five consecutive cells each from the exodermis and endodermis were randomly selected from the same root cross-section. A line was drawn across the cell membrane on both the distal and proximal sides of the cell, and the maximum signal intensity along each line was recorded. Finally, the signal intensities were calculated and presented based on measurements from 55 to 85 exodermal and endodermal cells, respectively.

### Suberin staining

Suberin staining was performed with Fluorol Yellow 088 according to Wang *et al*. (2019). Root cross-sections at 20 mm from the root apex were subjected to immunostaining as described above, followed by suberin staining by incubating in a freshly prepared solution of Fluorol Yellow 088 [Sigma; 0.01% (w/v) in lactic acid] at 70 °C for 30 min. The specimens were then rinsed with water three times at 70 °C, placed on a glass slide, and observed by confocal scanning microscopy (TCS SP8x, Leica Microsystems). To image Fluorol Yellow 088, excitation at 405 nm was used, and the signal was detected at 510–525 nm. The images of immunostaining and suberin staining were merged using the comparison of light and dark synthesis in GIMP (GNU image manipulation program version 2.10.38; https://www.gimp.org/).

### Statistical analysis

ANOVA, followed by Tukey–Kramer’s test, was used for comparison with the software BellCurve for Excel (Social Survey Research Information Co., Ltd).

## Results

### Phenotypic analysis of *oscasp1* mutants at different Mn concentrations

**Fig. 1.**
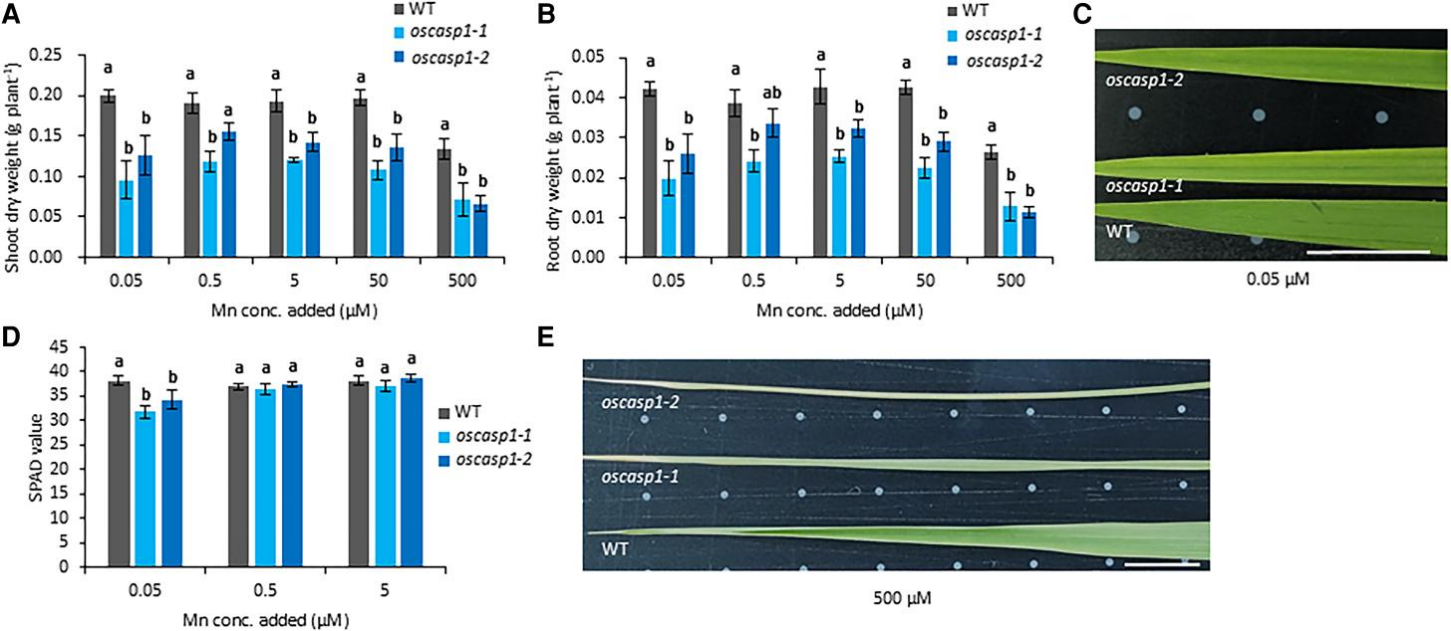
Phenotypic analysis of *oscasp1* mutants at different Mn concentrations. (A, B) Dry weight of shoots (A) and roots (B) of the WT and two independent *OsCASP1* knockout lines grown at different Mn concentrations. Seedlings (15 d old) were grown in a nutrient solution containing 0.05, 0.5, 5, 50, or 500 µM Mn for 16 d. (C) Phenotype of the leaf 5 in the WT and two independent *OsCASP1* knockout lines grown at 0.05 µM Mn. (D) The SPAD value of leaf 5 in the WT and *oscasp1* mutants grown at 0.05, 0.5, and 5 µM Mn. (E) Phenotype of leaf 6 in the WT and *oscasp1* mutants grown at 500 µM Mn. The data are presented as means ±SD (*n*=3–4). Significant differences were determined by Tukey–Kramer’s test and labeled with different letters (*P*<0.05).

To investigate the role of the endodermal CS in Mn uptake, two independent rice mutants defective in endodermal CS formation, *oscasp1-1* and *oscasp1-2*, and their WT (Wang *et al.*, 2019) were grown in a nutrient solution containing different Mn concentrations ranging from 0.05 µM to 500 µM. Overall, the growth of both the shoots and roots of the mutants was decreased compared with the WT at all Mn concentrations tested (Fig. 1A, B). This decrease has been attributed mainly to the overaccumulation of Ca (Supplementary Fig. S1; Wang *et al.*, 2019). However, in addition to these growth differences, we found chlorosis symptoms in the new leaves of mutants but not in the same leaves of the WT at 0.05 µM Mn (Fig. 1C). When the Mn supply was increased to >0.5 µM, this symptom in the mutants disappeared. Measurement of the SPAD value of leaf 5 showed that the value was significantly lower in the mutants than in the WT at 0.05 µM Mn, but not at 0.5 µM and 5 µM Mn (Fig. 1D).

On the other hand, at a high Mn concentration (500 µM), the growth of both shoots and roots was inhibited compared with other Mn concentrations in both the *oscasp1* mutants and the WT (Fig. 1A, B). Leaf curling was observed in leaf 6 of the mutants and WT, but obviously mutants showed more severe symptoms (Fig. 1E).

### Comparison of Mn concentration in the shoots and roots between the wild type and *oscasp1* mutants

**Fig. 2.**
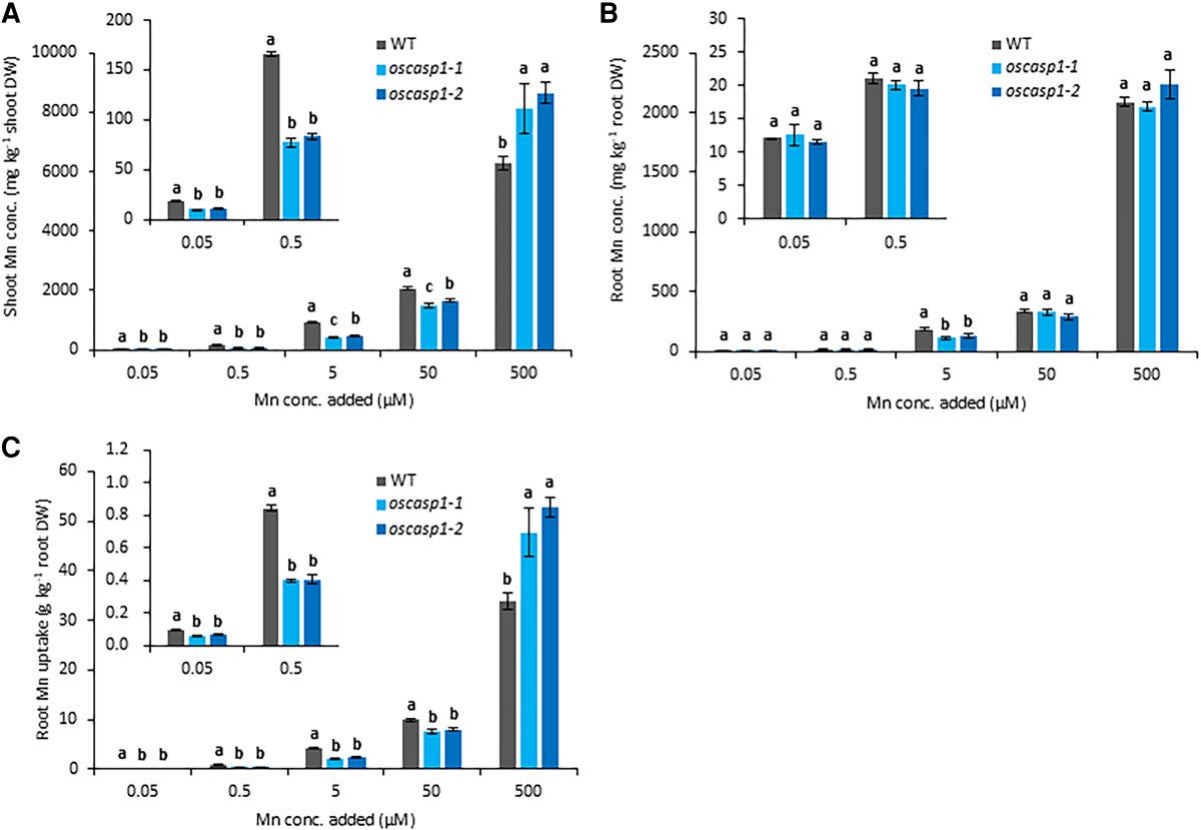
Mn concentration in the shoot and root of the WT and *oscasp1* mutants. (A, B) Mn concentration in the shoots (A) and roots (B) of the WT and *oscasp1* mutants. (C) Mn uptake. Seedlings (15 d old) were grown in a nutrient solution containing 0.05, 0.5, 5, 50, or 500 μM Mn for 16 d. The Mn concentration was determined by ICP-MS. The data are presented as means ±SD (*n*=3). Significant differences were determined by Tukey–Kramer’s test and labeled with different letters (*P*<0.05).

We compared the Mn concentrations in the shoots and roots of the WT and *oscasp1* mutants grown under different Mn supply conditions. The Mn concentration in the roots and shoots increased with increasing Mn concentrations in the nutrient solution in both *oscasp1* mutants and the WT (Fig. 2A, B). However, a comparison between the WT and mutants revealed opposite trends depending on the Mn concentration. At Mn supply <50 µM, the Mn concentration in the shoots of *oscasp1* mutants was significantly lower than that in the WT (Fig. 2A). However, at a high Mn concentration (500 µM), the shoot Mn concentration of the *oscasp1* mutants was higher than that of the WT (Fig. 2A). By contrast, there was no difference in the root Mn concentration between the mutants and WT at either Mn concentration, except a slight decrease in the mutants at 5 µM Mn (Fig. 2B). The Mn uptake calculated was higher in the WT than in the mutants at Mn concentrations <50 µM, but lower at 500 µM Mn (Fig. 2C).

We also compared other mineral element profiles of the shoots and roots between the WT and *oscasp1* mutants grown at different Mn concentrations. Regardless of the different Mn supply conditions, the Ca and Sr concentrations in the shoots of *oscasp1* were significantly higher than those of the WT (Supplementary Fig. S1), while the concentrations of Fe, Zn, Cd, Ge, K, and Rb in the shoots of the *oscasp1* mutants were lower compared with the WT. In particular, the concentrations of Fe, Zn, Cd, and Ge in the shoots of the *oscasp1* mutants were significantly decreased compared with the WT (Supplementary Fig. S1). By contrast, there was no significant difference in the concentrations of Mg, Cu, and P in the shoots between the *oscasp1* mutants and WT (Supplementary Fig. S1). In the roots, there was no large difference in the concentration of most mineral elements between the *oscasp1* mutants and the WT compared with large difference in the shoots (Supplementary Fig. S2). These results are almost consistent with those reported previously at 0.5 µM Mn (Wang *et al.*, 2019).

### Comparison of Mn concentration in xylem sap between *oscasp1* mutants and the wild type

**Fig. 3.**
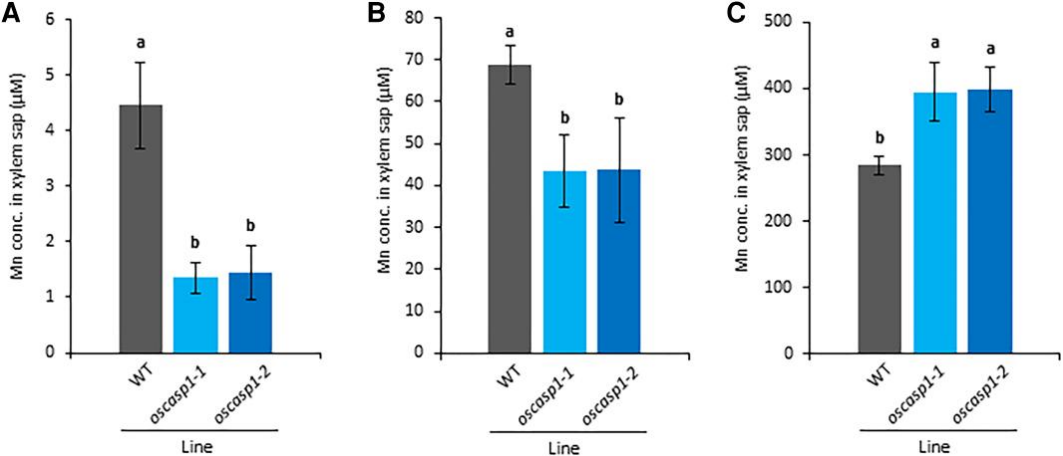
Comparison of Mn concentration in xylem sap of *oscasp1* mutants and the WT at different Mn concentrations. (A–C) Mn concentration in xylem sap of the WT and *oscasp1* mutants. Xylem sap was collected from the WT and *oscasp1* mutants (40 d old) exposed to different Mn concentrations including 0.5 (A), 50 (B), and 500 µM (C) for 6 h. The data are presented as means ±SD (*n*=4). Significant differences were determined by Tukey–Kramer’s test and labeled with different letters (*P*<0.05).

We further compared the Mn concentration in the xylem sap between the *oscasp1* mutants and WT at three Mn concentrations. The results showed that Mn concentration in the xylem sap was lower in the mutants than in the WT at 0.5 µM and 50 µM Mn (Fig. 3A, B). However, at 500 µM Mn, the Mn concentration in the xylem sap was higher in the mutants than in the WT (Fig. 3C). These results are consistent with the shoot Mn concentrations (Fig. 2A), supporting that Mn uptake was altered in the mutants.

### Comparison of expression of Mn transporter genes at different Mn concentrations

**Fig. 4.**
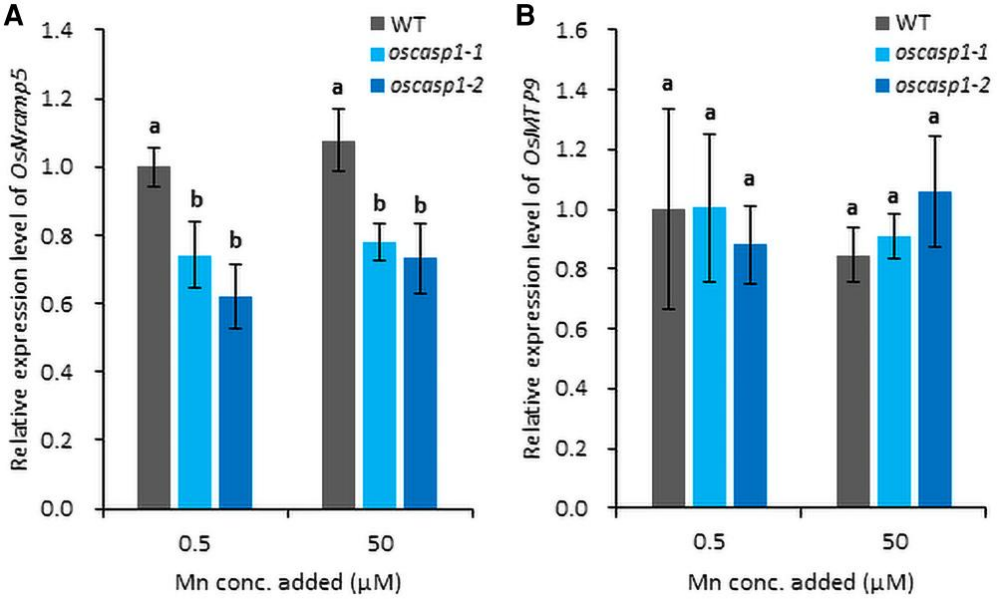
Gene expression analysis of *OsNramp5* and *OsMTP9* in *oscasp1* mutants and the WT under different Mn concentrations. (A) Expression of *OsNramp5* in the roots of the WT and *oscasp1* mutants. (B) Expression of *OsMTP9* in the roots of the WT and *oscasp1* mutants. WT and *oscasp1* mutants (16 d old) were treated with different Mn concentrations (0.5 µM or 50 µM) for 24 h before being subjected to RNA extraction. The data are presented as means ±SD (*n*=4). Significant differences were determined by Tukey–Kramer’s test and labeled with different letters (*P*<0.05).

To examine the mechanism behind decreased Mn uptake in *oscasp1* mutants (Fig. 2A, C), we compared the gene expression levels of two major transporter genes (*OsNramp5* and *OsMTP9*) involved in Mn uptake in the roots between the WT and *oscasp1* mutants at two different Mn concentrations (0.5 µM and 50 µM). The expression level of *OsNramp5* was unaffected by different Mn concentrations (Fig. 4A), while the expression level in the mutants was decreased by 31% compared with the WT regardless of the Mn concentrations (Fig. 4A). The expression level of *OsMTP9* did not differ between the WT and *oscasp1* mutants, and between low and high Mn concentration conditions in both lines (Fig. 4B).

### Quantitative analysis of OsNramp5 and OsMTP9 protein levels in *oscasp1* mutants

**Fig. 5.**
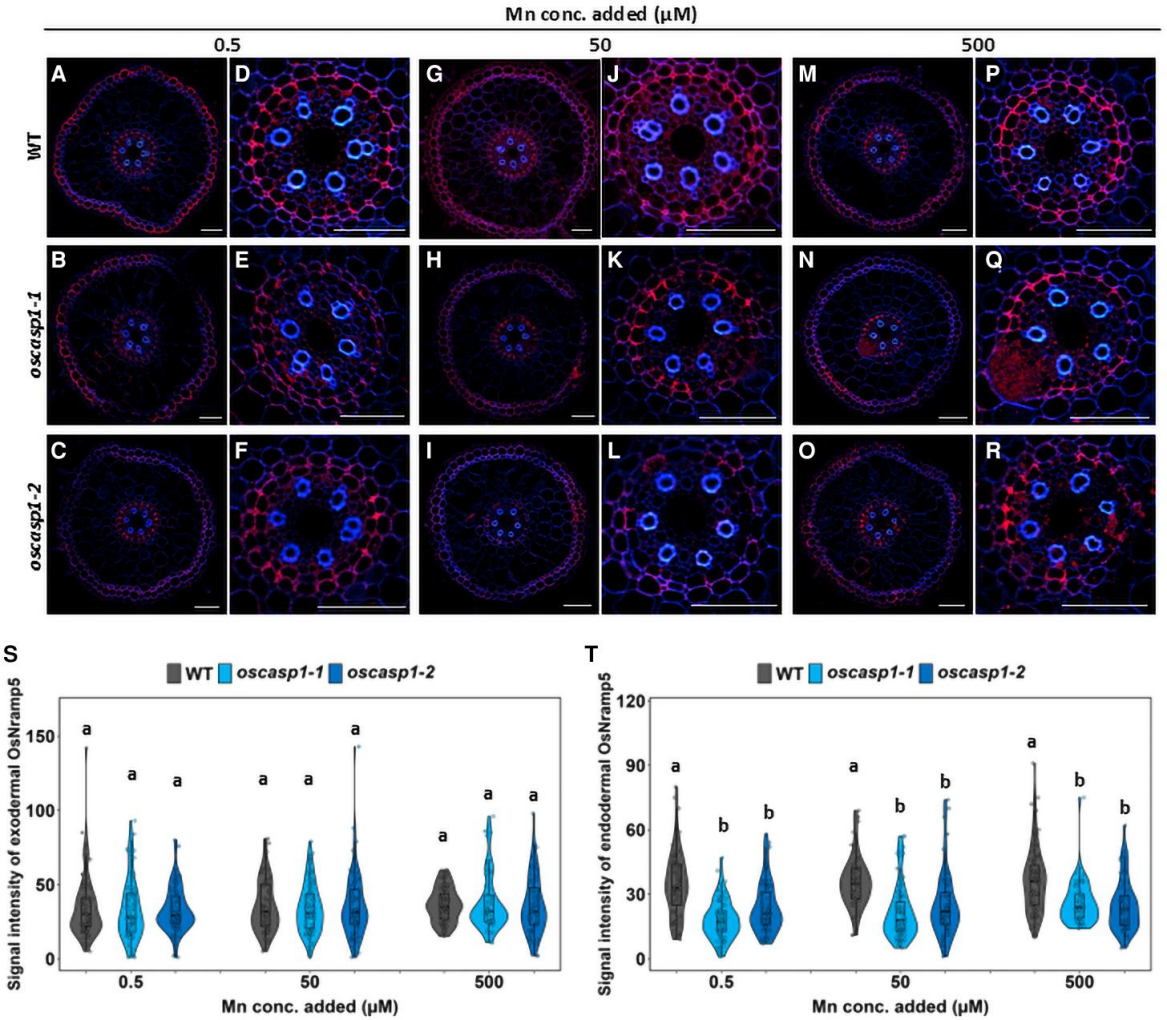
Cellular localization of OsNramp5 and its protein abundance in the roots of *oscasp1* mutants and the WT. (A–C, G–I, and M–O) Cellular localization of OsNramp5 in the roots of the WT (A, G, M) and *oscasp1* mutants (B–C, H–I, and N–O) grown at 0.5 µM (A–C), 50 µM (G–I), and 500 µM Mn (M–O). Scale bar=50 µm. (D–F, J–L, and P–R) Enlarged image of the endodermis in (A–C, G–I, and M–O). Scale bar=50 µm. (S, T) Quantitative analysis of OsNramp5 signal intensity in the exodermis (S) and endodermis (T) of the WT and *oscasp1* mutants. The WT and *oscasp1* mutants (6 d old) were exposed to different Mn concentrations (0.5, 50, or 500 µM) for 6 h. Immunostaining was performed using the seminal root cross-section (20 mm from the apex). Red color shows the signal from OsNramp5 and blue color that from cell wall autofluorescence. The data are presented as means ±SD (*n*=55∼80). Significant differences were determined by Tukey–Kramer’s test and labeled with different letters (*P*<0.01).

We further examined the protein levels of OsNramp5 and OsMTP9 in the roots of the WT and *oscasp1* mutants under different Mn concentration conditions (0.5, 50, and 500 µM). Similar to previous results (Sasaki *et al.*, 2012), OsNramp5 was polarly localized at the distal side of the exodermis and endodermis in the WT (Fig. 5A–R), and this polar localization was not altered in the mutants. Quantitative analysis of the signal intensity revealed that there was no difference in the OsNramp5 protein level at the exodermis between *oscasp1* mutants and the WT at all Mn concentrations tested (Fig. 5A–C, G–I, M–O, S). However, the signal intensity at the endodermis was significantly reduced in the *oscasp1* mutants compared with the WT (Fig. 5D–F, J–L, P–R, T), although the intensity was not affected by Mn concentrations.

**Fig. 6.**
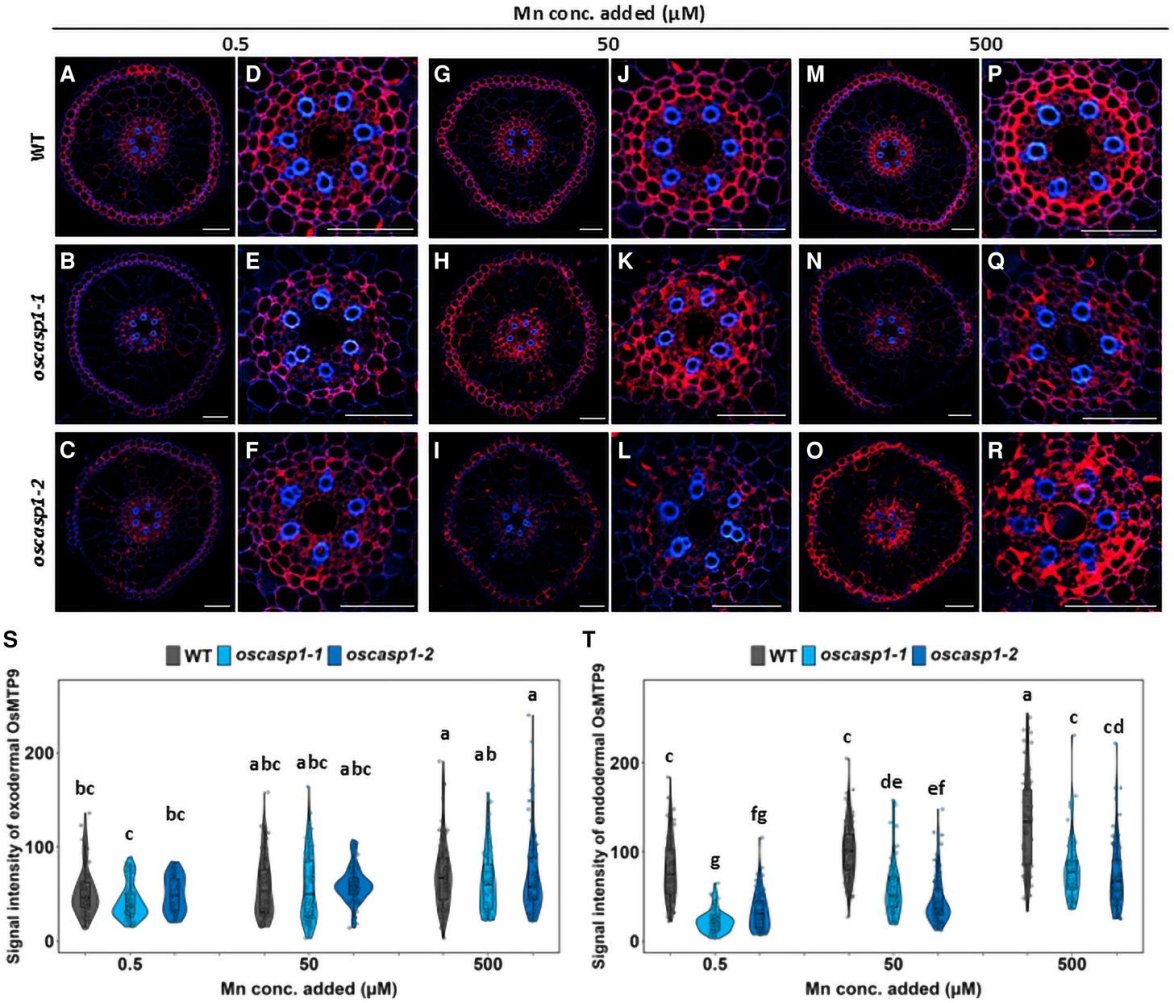
Cellular localization of OsMTP9 and its protein abundance in the roots of *oscasp1* mutants and the WT. (A–C, G–I, and M–O) Cellular localization of OsMTP9 in the roots of the WT (A, G, and M) and *oscasp1* mutants (B–C, H–I, and N–O) grown at 0.5 µM (A–C), 50 µM (G–I), and 500 µM Mn (M–O). Scale bar=50 µm. (D–F, J–L, and P–R) Enlarged image of endodermis in (A–C, G–I, and M–O). Scale bar=50 µm. (S, T) Quantification of OsMTP9 signal intensity in the exodermis (S) and endodermis (T) of the WT and *oscasp1* mutants. The WT and *oscasp1* mutants (6 d old) were exposed to different Mn concentrations (0.5, 50, and 500 µM) for 6 h. Immunostaining was performed using the seminal root cross-section (20 mm from the apex). Red color shows the signal from OsMTP9 and blue color that from cell wall autofluorescence. The data are presented as means ±SD (*n*=60–85). Significant differences were determined by Tukey–Kramer’s test and labeled with different letters (*P*<0.01).

The polar localization of OsMTP9 at the proximal side of both the exodermis and endodermis was also not altered in the mutants (Fig. 6A–R). The signal intensity of OsMTP9 at the exodermis did not differ between the *oscasp1* mutants and WT, but that at the endodermis was decreased in the mutants compared with the WT at all Mn concentrations tested (Fig. 6).

The signal intensity in both the WT and mutants was higher at higher Mn concentrations than at low Mn concentrations. This result is consistent with previous findings (Ueno *et al.*, 2015), although the exact mechanism is unknown.

### Suberin deposition pattern in roots of *oscasp1* mutants and the wild type

**Fig. 7.**
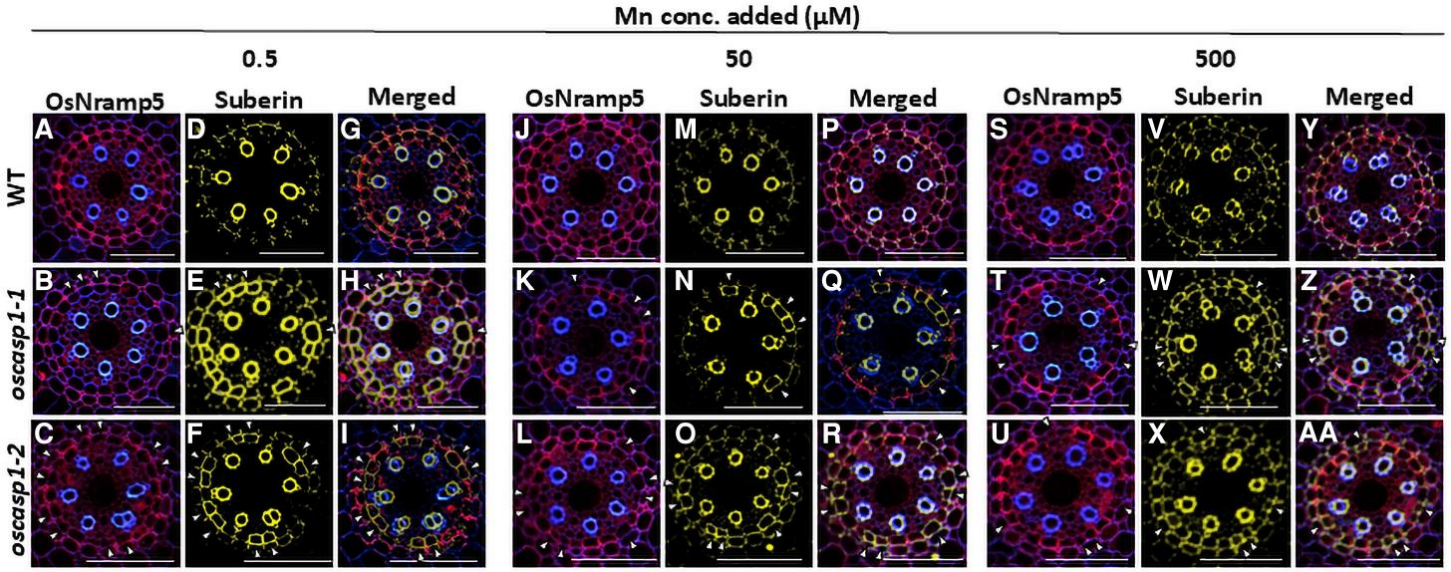
Localization of Mn transporter OsNramp5 and suberin in the roots of *oscasp1* mutants and the WT. (A–C, J–L, and S–U) Localization of OsNramp5 at the endodermis of the WT (A, J, and S) and *oscasp1* mutants (B–C, K–L, and T–U). (D–F, M–O, and V–X) Suberin deposition at the endodermis of the WT (D, M, V) and *oscasp1* mutants (E–F, N–O, and W–X). (G–I, P–R, and Y–AA) Merged image of OsNramp5 and suberin of the WT (G, P, and Y) and *oscasp1* mutants (H–I, Q–R, and Z–AA). Scale bar=50 µm. WT and *oscasp1* mutants (6 d old) were exposed to different Mn concentrations (0.5, 50, or 500 µM) for 6 h. Immunostaining was performed using the seminal root cross-section (20 mm from the apex), followed by suberin staining using the same root cross-section. Red color shows the signal from OsNramp5 and yellow color that from suberin. White arrows indicate endodermal cells where transporters are not localized, while suberin is overaccumulated.

**Fig. 8.**
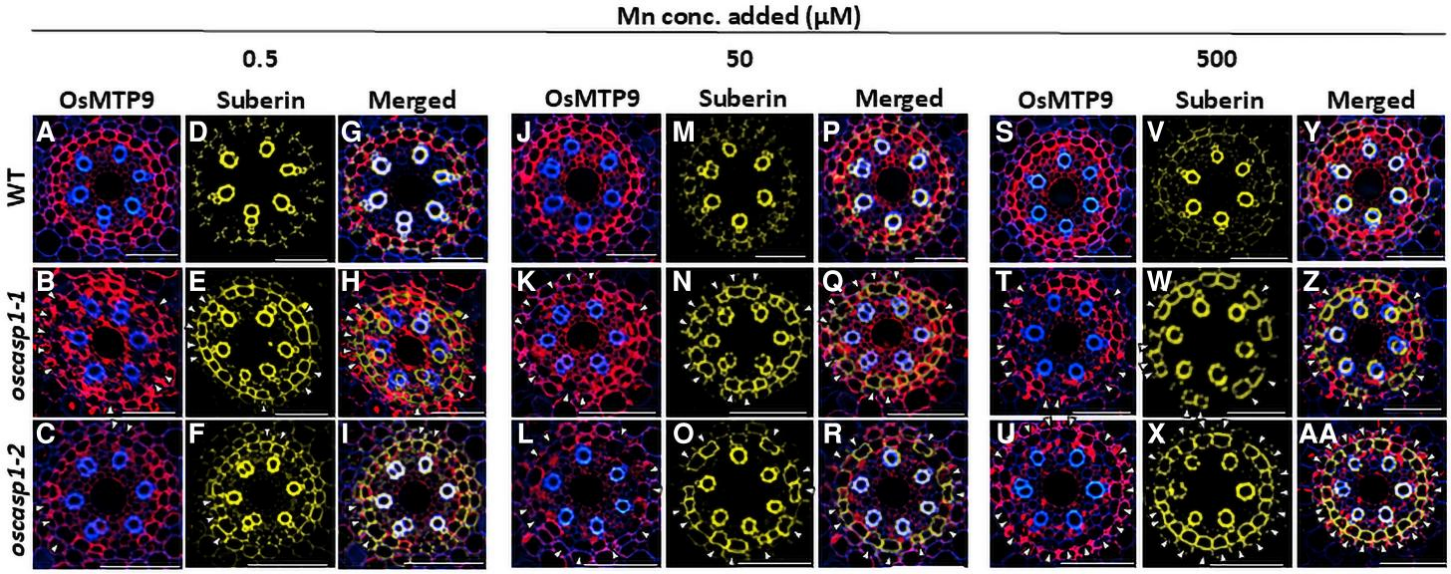
Localization of Mn transporter OsMTP9 and suberin in the roots of *oscasp1* mutants and the WT. (A–C, J–L, and S–U) Localization of OsMTP9 at the endodermis of the WT (A, J, and S) and *oscasp1* mutants (B–C, K–L, and T–U). (D–F, M–O, and V–X) Suberin deposition at the endodermis of the WT (D, M, and V) and *oscasp1* mutants (E–F, N–O, and W–X). (G–I, P–R, and Y–AA) Merged image of OsMTP9 and suberin of the WT (G, P, and Y) and *oscasp1* mutants (H–I, Q–R, and Z–AA). Scale bar=50 µm. WT and *oscasp1* mutants (6 d old) were exposed to different Mn concentrations (0.5, 50, or 500 µM) for 6 h. Immunostaining was performed using the seminal root cross-section (20 mm from the apex), followed by suberin staining using the same root cross-section. Red color shows the signal from OsMTP9 and yellow color that from suberin. White arrows indicate endodermal cells where transporters are not localized, while suberin is overaccumulated.

Immunostaining revealed that OsNramp5 and OsMTP9 showed patchy localization at the endodermis only in *oscasp1* mutants (Figs 5, 6), which may result in decreased abundance of these proteins (Figs 5, 6). Since a previous study reported that suberin is overaccumulated at the endodermis of *oscasp1* mutants (Wang *et al.*, 2019), we therefore compared the suberin deposition pattern with the transporter localization using the same tissues. Similar to previous findings (Wang *et al.*, 2019), we also observed overaccumulation of suberin at the endodermis of the mutants compared with the WT (Fig. 7). Merging suberin deposition with transporter localization showed that the signal of both OsNramp5 and OsMTP9 was not observed in endodermal cells with suberin deposition at different Mn concentrations (Figs 7, 8). However, the signal of transporters was observed at the endodermal cells without suberin deposition (Figs 7, 8). These results indicate that Mn transporter localization and suberin deposition are mutually exclusive.

## Discussion

Rice is able to grow under both upland and submerged conditions, where Mn concentration in soil solution differs greatly (>100-fold) (Sasaki *et al.*, 2011). To cope with large great fluctuations in Mn concentration, rice has developed a sophisticated system for Mn homeostasis by regulating transporters involved in uptake, distribution, and detoxification at different organs (Shao *et al.*, 2017). In terms of uptake, Mn uptake in rice roots is mainly mediated by two transporters; OsNramp5 and OsMTP9 (Sasaki *et al.*, 2012; Ueno *et al.*, 2015). Both *OsNramp5* and *OsMTP9* are highly expressed in the mature root region; however, their expression is not affected by different Mn concentrations. Furthermore, these transporters show polar localization at the distal and proximal side, respectively, of the exodermis and endodermis, forming a directional and efficient uptake system for Mn (Shao *et al.*, 2017). Since in the root mature region in rice, aerenchyma is well developed, Mn in soil solution is first transported to the apoplastic space of aerenchyma by OsNramp5–OsMTP9 at the exodermis, followed by the same transporter pair at the endodermis toward the stele (Shao *et al.*, 2017). On the other hand, the CS is also located at the exodermis and endodermis of the root mature region of rice (Enstone *et al.*, 2002), where Mn transporters are localized (Sasaki *et al.*, 2012; Ueno *et al.*, 2015). It was reported that some rice mutants defective in CS formation showed lower Mn uptake under normal Mn concentration (Wang *et al.*, 2019, 2022), implying the important role of the CS in Mn uptake in addition to Mn transporters. However, it is unclear whether the CS plays the same role in Mn uptake at different Mn concentrations and whether there is any relationship between the CS and transporters localized in the same cell layer. In the present study, through detailed analysis using *oscasp1* mutants, we found that the CS plays different roles in Mn uptake depending on Mn concentrations in the external solution. At low Mn concentration, the defect of the CS decreased Mn uptake in *oscasp1* mutants, whereas at high Mn concentration, the defect of CS increased Mn uptake (Fig. 3).

Mn uptake by rice roots mainly relies on transporters, especially at low Mn concentrations. This is demonstrated by the finding that knockout of *OsNramp5* resulted in almost complete loss of Mn uptake at low Mn concentrations (<5 µM) (Sasaki *et al.*, 2012). In the *oscasp1* mutants, we found that the defect of the endodermal CS resulted in decreased protein levels of two Mn transporters (OsNramp5 and OsMTP9) at the endodermis, but not at the exodermis (Figs 5, 6). Furthermore, we found that the transporters were not expressed in the endodermal cells with suberin deposition (Figs 7, 8). Although there was no difference in suberin deposition in the exodermis between the WT and *casp1* mutant (Wang *et al.*, 2019), knockout of *OsCASP1* caused early suberization at the endodermal cells (Wang *et al.*, 2019), suggesting that suberin deposition at the endodermal cells affects the expression of Mn transporters in the *oscasp1* mutants. The effect of suberin deposition on mineral element uptake was also previously reported in Arabidopsis and rice. For example, among Arabidopsis mutants with defective endodermal CS formation, those with overaccumulation of suberin, including *esb1*, *casp1-1 casp3-1*, *myb36*, and *lotr1*, showed reduced Mn uptake (Hosmani *et al.*, 2013; Kamiya *et al.*, 2015; Li *et al.*, 2017), while those without suberin deposition such as *sgn3* showed a similar Mn uptake to the WT (Pfister *et al.*, 2014). Arabidopsis *myb1 mtb53 myb92 myb93* quadruple mutants with lower suberin deposition showed higher Mn in the leaves (Shukla *et al.*, 2021). Similarly, rice mutants, including *casp1*, *gapless1*, *gapless2/3*, *gapless1/2*, and *myb36a*, with suberin deposition showed lower Mn accumulation in the shoots (Hosmani *et al.*, 2013; Kamiya *et al.*, 2015; Li *et al.*, 2017; Wang *et al.*, 2019, 2022; Song *et al.*, 2023). Although in these studies, the mutually exclusive relationship between suberin deposition and transporter expression was not investigated, there is a possibility that like *oscasp1* investigated in this study, suberin deposition at the endodermal cells affects the expression of Mn transporters (Figs 7, 8). Suberin is a hydrophobic polymer composed mainly of long-chain fatty acids with glycerol and aromatic compounds. It covers the entire endodermal cells, which was proposed to prevent access to the transporters localized at the plasma membrane (Geldner, 2013; Barberon and Geldner, 2014). However, in the present study, we found that suberin deposition suppresses the expression of the transporters for Mn uptake in rice (Figs 7, 8), although the underlying mechanism needs to be investigated in the future. In a recent study, protein abundance of an Si transporter, Lsi1 (Ma *et al.*, 2006), was also reduced in an OsMYB92a-overexpressing line with increased suberin deposition (Chen *et al.*, 2024), suggesting that many uncharacterized transporters are suppressed by suberin deposition at the endodermal cells. The Cd uptake was also significantly reduced in the mutants. This is because similar to Mn, Cd uptake is also mediated by OsNramp5 (Ishimaru *et al.*, 2012; Sasaki *et al.*, 2012). In addition, the endodermal CS may also be important to prevent backflow of Mn from the stele to the aerenchyma at low Mn concentrations.

There are two possibilities for the suberin-suppressed expression of Mn transporters. One is that suberin biosynthesis and transporter expression may share the same signaling pathway, such that activation of one inhibits the other. The other one is that the excessive suberin accumulation may physically hinder the proper localization or stability of transporters at the plasma membrane. However, the exact mechanisms need to be examined in the future.

At a high Mn concentration (500 µM), although the abundance of both OsNramp5 and OsMTP9 at the endodermis was also decreased in the *oscasp1* mutants (Figs 5, 6), the Mn uptake was not decreased but rather increased in the mutants compared with the WT (Fig. 3C). This increase could be attributed to uncontrolled diffusion of high Mn from aerenchyma to the stele due to the defect of CS at the endodermal cells, indicating that the endodermal CS functions as an apoplastic barrier for Mn. Similarly, in Arabidopsis, it was reported that the endodermal CS plays an important role in preventing excess B to the stele (Muro *et al.*, 2023).

In conclusion, our results reveal that the defect of endodermal CS in *oscasp1* mutants showed different effects on Mn uptake depending on the external Mn concentrations. At low Mn concentrations, compensatory suberin deposition at the endodermal cells suppresses the expression of transporters (OsNramp5 and OsMTP9) for Mn uptake, thereby decreasing Mn uptake. By contrast, at high Mn concentration, endodermal CS plays an important role in preventing excess Mn into the stele by restricting its apoplastic transport in rice.

## Supplementary Material

eraf302_Supplementary_Data

## Data Availability

The data supporting the findings of this study are available within the manuscript and its Supplementary data.

## Supplementary data

The following supplementary data are available at *JXB*  online.

Fig. S1. Mineral element concentration in the shoots of the wild type (WT) and *oscasp1* mutants grown at different Mn concentrations.

Fig. S2. Mineral element concentration in roots of the wild type (WT) and *oscasp1* mutants grown at different Mn concentrations.
