# Supplementary material for: Dual roles of suberin deposition at the endodermal Casparian strip in manganese uptake of rice
Source: J Exp Bot. 2025 Jul 7;76(20):6064–74. doi: 10.1093/jxb/eraf302 (PMC12621096; doi:10.1093/jxb/eraf302)
Supplement: eraf302_Supplementary_Data [file eraf302_supplementary_data.pdf]

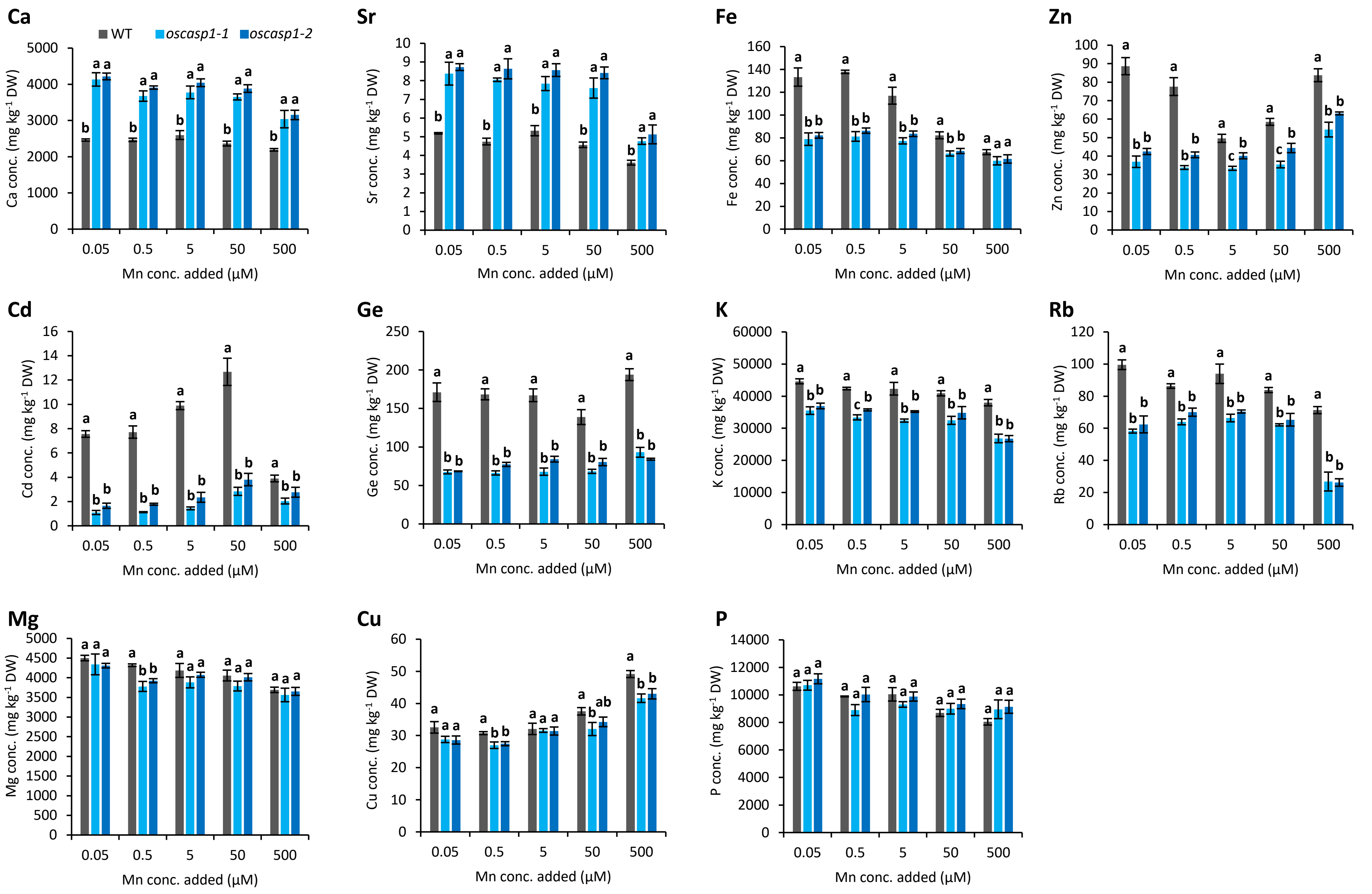

Supplementary Fig. S1. Mineral element concentration in the shoots of wild type (WT) and *oscasp1* mutants grown at different Mn concentrations. Concentration of Ca, Sr, Fe, Zn, Cd, Ge, K, Rb, Mg, Cu and P in shoots of WT and *oscasp1* mutants. After the seedlings (15-day-old) of both WT and *oscasp1* mutants were grown in a nutrient solution containing 0.05, 0.5, 5, 50, 500 μM Mn for 16 d, the plants were exposed to a nutrient solution containing 1 μM of Sr, Rb, Ge, and Cd for 1 d before harvest. The shoots were harvested for mineral element analysis by ICP-MS. The data are presented as means ± SD (n=3). Significant differences were determined by Tukey–Kramer's test and labeled with different letters (P<0.05).

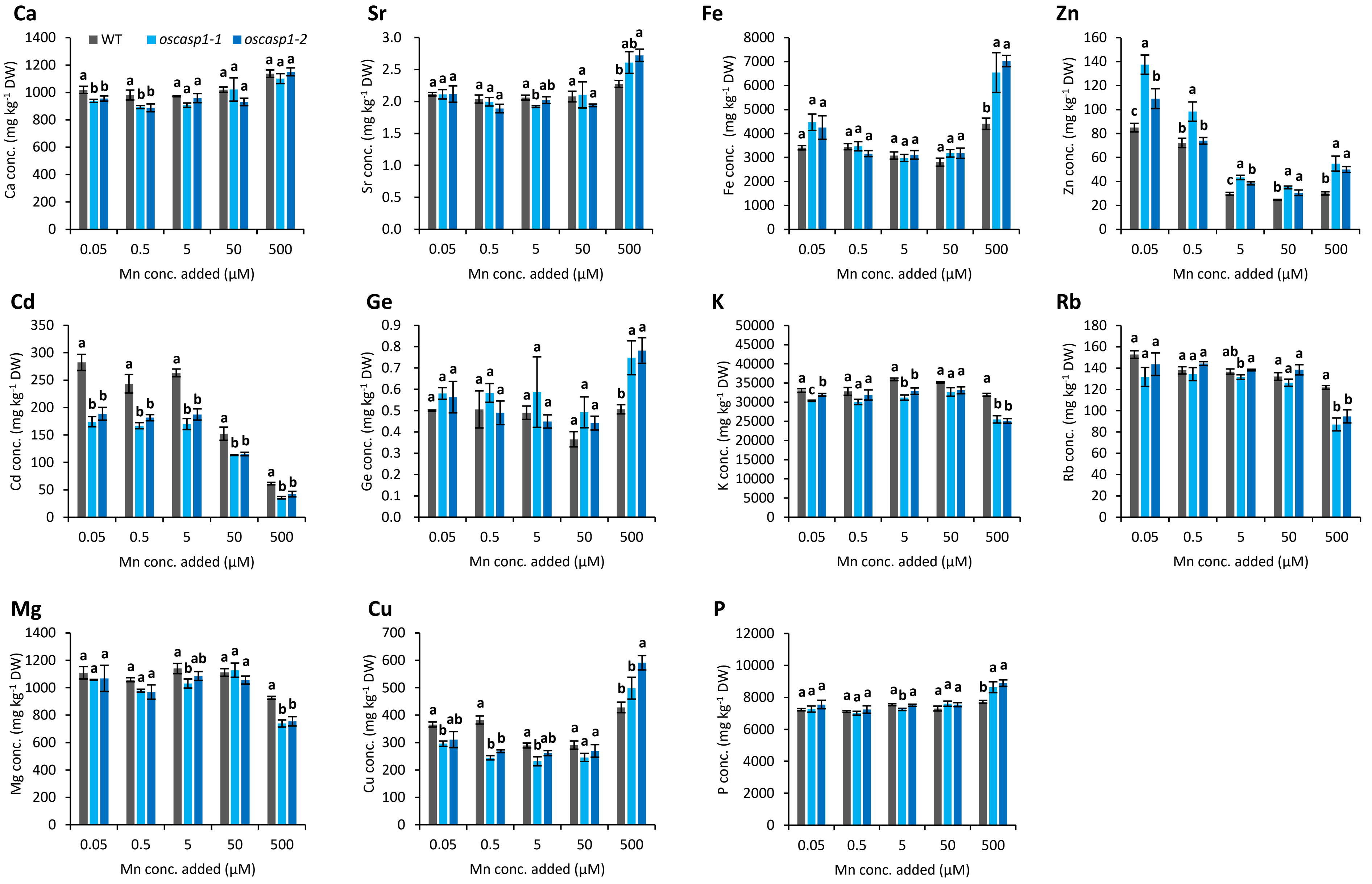

Supplementary Fig. S2. Mineral element concentration in roots of wild type (WT) and *oscasp1* mutants grown at different Mn concentrations. Concentration of Ca, Sr, Fe, Zn, Cd, Ge, K, Rb, Mg, Cu and P in roots of WT and *oscasp1* mutants. After the seedlings (15-day-old) of both WT and *oscasp1* mutants were grown in a nutrient solution containing 0.05, 0.5, 5, 50, 500 μM Mn for 16 d, the plants were exposed to a nutrient solution containing 1 μM of Sr, Rb, Ge, and Cd for 1 d before harvest. The roots were harvested for mineral element analysis by ICP-MS. The data are presented as means ±SD (n=3). Significant differences were determined by Tukey–Kramer's test and labeled with different letters (P<0.05).
